# Evaluation of digital PCR assay in detection of *M.tuberculosis* IS6110 and IS1081 in tuberculosis patients plasma

**DOI:** 10.1186/s12879-020-05375-y

**Published:** 2020-09-07

**Authors:** Lingna Lyu, Zihui Li, Liping Pan, Hongyan Jia, Qi Sun, Qiuyue Liu, Zongde Zhang

**Affiliations:** Beijing Key Laboratory for Drug Resistant Tuberculosis Research, Beijing Tuberculosis and Thoracic Tumor Research Institute, Beijing Chest Hospital, Capital Medical University, 97 Machang Road, Tongzhou District, Beijing, 101149 China

**Keywords:** Tuberculosis, Digital PCR, Cell-free DNA, IS6110, IS1081

## Abstract

**Background:**

Tuberculosis is still a significant diagnostic and therapeutic challenge with high proportion of smear- and culture- negative incidences worldwide. The conventional diagnostic tests are time-consuming and have a low sensitivity. Digital PCR is a novel technology which can detect target sequences with relatively low abundance and obtain the absolute copy numbers of the targets.

**Methods:**

We assessed the accuracy of dPCR in TB diagnosis using more than 250 specimens, and for the first time, we selected *M.tuberculosis*-specific IS1081 in addition to widely used IS6110 as the amplification targets for dPCR. The quantification of target DNA was calculated using QuantaSoft Version 1.7.4.0917 (BioRad), and SPSS version 13.0 software (SPSS Inc., Chicago, IL, USA) was used for statistical analyses.

**Results:**

IS6110-dPCR was more sensitive than IS1081, with the sensitivity and specificity accounting for 40.6 and 93.4% respectively. When we classified the TB patients by personal factors for high copy number of *M.tuberculosis* derived DNA in plasma: bilateral TB, extrapulmonary TB and disseminated TB, the sensitivity of both IS6110- and IS1081- dPCR was the highest in patients with disseminated TB (IS6110, 100%; IS1081, 68.8%), while their sensitivity was a bit higher in patients with extrapulmonary TB (IS6110, 50.0%; IS1081, 39.3%) than that in bilateral TB (IS6110, 43.3%; *IS1081*, 33.3%). Compared with traditional TB diagnostic tests, joint detection IS6110 & IS1081-dPCR was not as sensitive as smear microscope or mycobacterial culture, but it was higher than IS6110 qPCR (*p* < 0.05) and was able to detect 47.4% of smear-negative TB patients.

**Conclusion:**

Our study suggested that plasma IS6110-dPCR is a rapid, moderate accurate and less-invasive method to detect *M.tuberculosis* DNA in plasma of TB patients and IS6110 & IS1081-dPCR has a potential to aid diagnosis of smear-negative TB.

## Background

Tuberculosis (TB) is still one of the most infectious killers in the world, causing more death even than HIV and Malaria. In 2018, an estimated 9 million people were infected by *M.tuberculosis* and over 1 million died from TB [1]. Early accurate diagnosis of TB is extraordinarily important for TB treatment and disease control.

Mycobacterial culture is the gold standard for TB diagnosis, but not widely available due to technical and bio-safety requirements. Sputum smear microscopy is the most available and used TB diagnostic low- and middle-income countries, however, it was less sensitive because 5000–10,000 bacilli per mL of sputum were required for showing a positive result [2]. In recent years, nucleic acid amplification-based tests (NATs) for *M.tuberculosis* specific regions have been developed, such as conventional PCR, quantitative real-time PCR (qPCR), loop-mediated isothermal amplification (LAMP), as well as Xper MTB/RIF recommended by WHO [3–6]. Adopting those accurate and rapid molecular techniques, these platforms were widely used to detect *M.tuberculosis* in clinical samples. However, the accuracy of these techniques heavily relied on sufficient amount of samples although their requested quantity for the test was relatively low. For children, sputum-free or symptom less patients without respiratory lesions, the difficulty of access to obtain enough samples results in reduced sensitivity and accuracy of those diagnostic tests. Thus, developing new methods with high sensitivity and specificity from easily and stably available samples such as blood for the timely and accurate diagnosis was significant for TB control.

However, *M.tuberculosis* culture and relative quantification by PCR using blood samples have a fairly low positive rate. Recently, digital polymerase chain reaction (dPCR) has emerged as a novel nucleic acid quantitative technique which was carried out using a relatively small amount of target molecules. Compared to the most popular quantitative PCR (qPCR), dPCR presents absolute quantification without a standard curve [7]. Currently, several dPCR based platforms have been developed according to different sample dispersion ways, among which the water-in-oil droplet packaging fractionated samples was the most frequently used [8, 9]. Previous studies have applied dPCR in various fields including clinical pathogen detection, DNA methylation detection, prenatal diagnosis, circulating nucleic acid quantification and gene mutation analysis [10–14]. Our recent work also reported that dPCR was able to successfully detect cell-free *M.tuberculosis* DNA from CSF of TBM and had the potential to enhance the diagnosis of TBM [15]. Moreover, there were several initial researches that had successfully detected *M.tuberculosis* derived nucleic acids in TB patients’ plasma [16–18]. However, they worked on small scales of specimens (no more than 60 in total) although their results demonstrated dPCR was a potential diagnostic method with high specificity and sensitivity. The performance of dPCR testing in a larger sample size of TB specimens was an urgent need for its clinical application.

To address these concerns, we conducted a study on the diagnostic accuracy of dPCR assay using IS6110, and for the first time, also using IS1081, as targets to detect *M.tuberculosis* DNA in plasma of TB on a larger scale of specimens. Our results presented here confirmed the potential use of dPCR as a rapid and sensitive molecular test for TB diagnosis.

## Methods

### Clinical specimens

A total of 261 plasma samples from clinical patients were consecutively enrolled from March 2013 to October 2015 in this study. All the pulmonary TB patients were identified based on positive mycobacterial culture and/or smear test results. All enrolled healthy control subjects were healthy workers from a physical examination program and they were confirmed not to have *M.tuberculosis* infection by normal computed tomography (CT) chest films, negative T-SPOT (Oxford Immunotec, Oxford, UK) results. The medical records were collected according to ages, gender, complications, underlying diseases as well as examination results. The protocol was approved by the Ethics Committee of the Beijing Chest Hospital, Capital Medical University.

### Categorization of patients

As bilateral lung distribution, extropulmonary lesions and disseminated bacteria into blood were identified as personal factors for high copy number of *M.tuberculosis* derived DNA in plasma [16], TB patients were divided into three groups according to clinical examination results: (1) Bilateral TB: patients were diagnosed to have *M.tuberculosis* lesions in both lungs by the findings from image examinations. (2) Extrapulmonary TB: patients were diagnosed by direct sampling or findings of image examinations in addition to pulmonary TB; (3) Disseminated TB: patients were identified according to isolation of *M. tuberculosis*, positive PCR, or histologic demonstration of caseating granulomatous inflammation from bone marrow, blood, liver biopsy specimen, or at least two noncontiguous organs with or without miliary lung lesions and isolation of *M. tuberculosis*, positive PCR, or histopathological identification of caseating granulomas from one organ and radiographic finding of miliary lung lesions. The three groups have overlapped TB patients.

### DNA extraction

Total DNA from 800 μl plasma was extracted using DNeasy Blood and Tissue Kits (QiaGen, Hilden, Germany) according to manufacturer’s instructions, but we eluted the DNA with 45 μl elution buffer so as to increase the DNA yield. The DNA samples were stored at − 20 °C until dPCR was performed.

### Droplet dPCR assay and data analysis

We used DNA insert sequences 6110 (IS6110) and 1081 (IS1081) as detection targets in our study, since they were both conserved in *M.tuberculosis* complexes. The PCR primers and internal probes (FAM labeled) of IS6110 were designed according to the reference [16], while the PCR primers and internal probes of IS1081(HEX labeled) were designed by using Primer Premier 5.0 software: Forward sense, 5′-CCTGCTGCACTCCATCTAC-3′, Reverse sense, 5′-CGTCGAGTACCCGATCATAT-3′, probes, 5′-[HEX]-CCCGACGCCGAATCAGTTGT-[BHQ-2]-3′. All the primers and probes were synthesized by Sangon Biotech (Shanghai, China). The PCR reaction formula and running profile were applied from what have described [15]. Briefly, PCR mix was composed of ddPCR™ Supermix for probes (1,863,010, BioRad, Hercules, CA, US), 0.9 μM primers, 125 nM probe and extracted DNA samples without dilution. The mixtures and droplet generation oil (1,863,005, BioRad) were added in cartridges and loaded into a QX200 droplet generator (BioRad) for droplet generation. After the droplet emulsions were transferred to a 96-well PCR plate and sealed with a foil heat seal, the PCR reactions were conducted at 95 °C for 10 min, 40 cycles of 94 °C for 30 s and 54 °C for 40 s, following a final cycle of 98 °C for 10 min. QX200 Droplet Reader (BioRad) was used to automatically measure the fluorescence signal of each droplet in the plate. The quantification of target DNA was calculated using QuantaSoft Version 1.7.4.0917 (BioRad) and presented as copy numbers per 20 μl of reaction mixture. Non-template negative control and *M.tuberculosis* H37Rv DNA positive control were adopted in all dPCR assays. Tests for each DNA sample were performed in duplicate. The threshold levels for selecting positive droplets were determined by the fluorescence intensities of the standard droplets applied to the k-nearest neighbor algorithm, “defifinetherain” [19].

### Statistic analysis

SPSS version 13.0 software (SPSS Inc., Chicago, IL, USA) was used for statistical analysis. Student’s *t*-test, Mann-Whitney *U* test or Wilcoxon test were applied to compare continuous variables, while Chi-Squared test or McNemar test were used to analyze categorical variables. Agreement of amplificated copy number between IS6110- and IS1081-dPCR was analyzed by Bland-Altman analysis [20]. A correlation study was performed with the Spearman’s rank correlation procedure (r). All *p*-values were two-sided, and values less than 0.05 were considered statistically significant.

## Results

### Patient characteristics

The demographic data for 261 subjects are shown in Table 1. A total of 155 pulmonary tuberculosis (PTB) patients were enrolled, including 111 Bilateral PTB, 56 PTB combined with Extrapulmonary TB, and 16 PTB combined with Disseminated TB. As control groups, 106 healthy subjects were recruited. Overall, the mean age for PTB and Healthy control (HC) were 39.9 (range from 20 to 79) and 24.1 (range from 18 to 44), while 63.2% (98/155) and 57.5% (61/106) were male in each group, respectively.

**Table 1:** Basic demographic and clinical features of participants

Characteristics	Total TB patients (*n* = 155)	Total HC (*n* = 106)
	Bilateral TB (*n* = 111)	–
	^a^ Extrapulmonary TB (*n* = 56) - tuberculous Pleurisy (*n* = 19) - lymphoid tuberculosis (*n* = 18) - bone tuberculosis(*n* = 8) - tuberculous meningitis (*n* = 6) - intestinal tuberculosis (*n* = 5)	–
	^a^ Disseminated TB (*n* = 16)	–
Median age ± SD (range)	39.9 ± 16.4 (20–79)	24.1 ± 6.9 (18–44)
Gender (male/female)	98/57	61/45
Smokers/Non-smokers	59/96	38/68

*TB* tuberculosis, *HC* healthy control

^a^, represents pulmonary tuberculosis associated with other disease features

### Results of dPCR for detecting *M.tuberculosis* DNA in plasma of patients

**Fig. 1:**
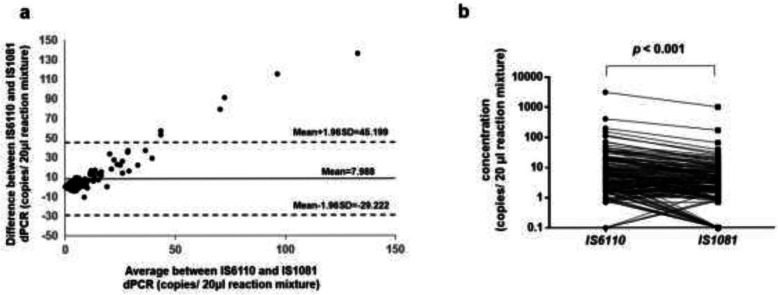
Duplex amplification of *M.tuberculosis* IS6110 and IS1081 DNA in plasma samples by dPCR. **a** Agreement of amplificated copy number between IS6110- and IS1081-dPCR plotted by Bland-Altman analysis. **b** The differences in copy number of IS6110- and IS1081-dPCR analyzed by Wilcoxon test

**Fig. 2:**
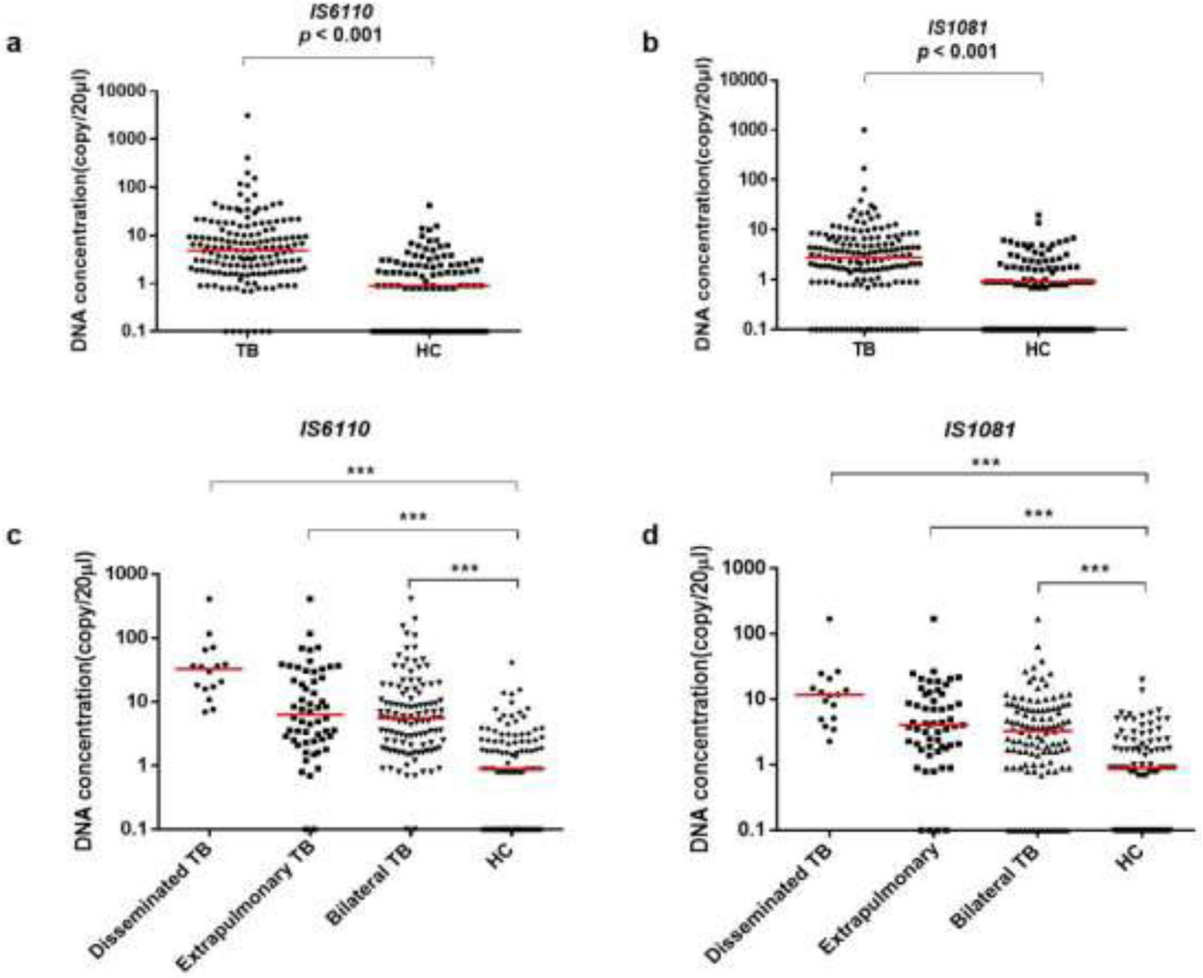
Quantification of *M.tuberculosis* IS6110 and IS1081 DNA in plasma samples by dPCR. **a, b** IS6110 and IS1081 copy number in plasma of total TB patients and HC individules, respectively (Mann-Whitney U test). **c, d** IS6110 and IS1081 copy number in plasma of Disseminated TB, Extrapulmonary TB, Bilateral TB and HC individuals, respectively (Mann-Whitney U test). Each dot represents the everage of duplicated dPCR results. Results were considered significant when *p* < 0.05

The target DNA concentrations are calculated based on Poisson distribution and some of the original data are listed in Fig. S1. Results of two repeated tests of 42 randomly selected plasma samples were highly correlated (IS6110, *r* = 0.803, *p* < 0.001; IS1081*, r* = 0.723, *p* < 0.001, Fig. S2). Agreement of amplificated copy number between IS6110- and IS1081- dPCR were analyzed and showed in Bland-Altman plot (Fig. 1a), which presented the average discrepancy (Mean) of 7.988 and more than 96% (149/155) plots lying within 95% limits of agreement from − 29.22 (Mean-1.96SD) to 45.20 (Mean + 1.96SD). Meanwhile, about 72.9% (113/155) IS6110- dPCR detected higher copy number of *M.tuberculosis* DNA in plasma of PTB patients than IS1081-dPCR for the same samples (*p* < 0.001, Fig. 1b). The copy number in 20 μl reaction mixture of tested PTB samples were significantly higher than that of HC group: median (minimum, maximum) for IS6110-dPCR was 4.9 (0.0–3150) vs 0.9 (0.0–42) with *p* < 0.001, and that for IS1081-dPCR was 2.8 (0.0–1001) vs 0.9 (0.0–20) with *p* < 0.001 (Fig. 2a and b). When we checked the results in the detailed groups of Bilateral TB, Extrapulmonary TB and Disseminated TB, the number of copies was remarkably higher in each of group than that in HC (Fig. 2c and d).

### Evaluation of diagnostic performance of dPCR for TB

**Fig. 3:**
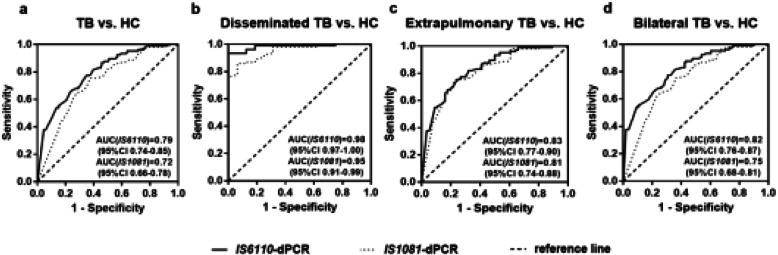
Diagnostic performance of *M.tuberculosis* IS6110- and IS1081-dPCR assay in TB. **a-d** Receiver-operating characteristic (ROC) curve of the ability to detect *M.tuberculosis* IS6110 and IS1081 DNA in the total TB, Disseminated TB, Extrapulmonary TB and Bilateral TB, respectivelly. AUC, area under ROC curve; Cl, confidence interval

Receiver operating characteristics (ROC) curve analysis was performed to evaluate the ability to detect *M.tuberculosis* DNA in plasma of PTB patients. The overall area under ROC curve (AUC) of IS6110-dPCR (0.79, 95% confidence interval (CI) 0.74–0.85) was slightly higher than that of IS1081-dPCR (0.72, 95% CI 0.66–0.78) (Fig. 3a). Notably, the AUC of dPCR in PTB patients with Disseminated TB was larger than that in Extrapulmonary TB and Bilateral PTB with IS6110-dPCR 0.98 (95% CI 0.97–1.00) and IS1081-dPR 0.95 (95% CI 0.91–0.99), respectively (Fig. 3b). The AUC for both IS6110- and IS1081- dPCR in patients with Extrapulmonary TB are almost the same: 0.83 (95% CI 0.77–0.90) and 0.81 (95% CI 0.74–0.88), respectively (Fig. 3c). However, for Bilateral PTB patients, the AUC of IS6110-dPCR (0.82, 95% CI 0.76–0.87) was larger than that of IS1081-dPCR (0.75, 95% CI 0.68–0.81) (*p* < 0.001, Fig. 3d).

The diagnostic performance of dPCR was presented in Table 2. Reading from the ROC curves, we set 7.0 (IS6110) and 5.5 (IS1081) copies per 20 μl reaction mixture as cut-off value to ensure high specificity as well as the largest sum of sensitivity and specificity. The sensitivity of IS6110-dPCR assay for total PTB was higher than that of IS1081-dPCR assay (40.6% vs. 27.1%, *p* < 0.001), while they had the same specificity (93.4%). The sensitivity of dPCR assay in defining Disseminated TB (IS6110, 100%; IS1081, 68.8%) was higher than that in Extrapulmonary TB (IS6110, 50.0%; *IS1081*, 39.3%) and Bilateral TB (IS6110, 43.3%; IS1081, 33.3%). Besides, when we reset the cut-off value with 16 (IS6110) and 14 (IS1081) copies per 20 μl reaction mixture, the specificity of dPCR assay was greatly increased to 99.1% for both targets but the sensitivity was compromised (IS6110, 77.8%; IS1081, 37.5%) (Table 3). The diagnostic performance of joint detection of IS6110 and IS1081 by dPCR (at least one of them was detected as positive on the basis of their cut-off value, notified as IS6110 & IS1081-dPCR, hereinafter) was also examined. We found that the sensitivity of the joint detection was improved compared with single target detection between classified groups of total PTB vs HC (from 40.6 to 42.6%), Extrapulmonary TB vs HC (from 50.0 to 51.8%) as well as Bilateral TB (from 43.2 to 45.9%) (Table S1).

**Table 2:** Diagnostic performance of plasma IS6110- and IS1081- dPCR assay for TB

	Cut-off point (copies/20 μl)	AUC	Sensitivity(%)	Specificity(%)	LR+	LR-	PPV (%)	NPV (%)
**Total TB (*n*** **= 155) compared with HC (*n*** **= 106)**								
IS6110-dPCR	7	0.79 (0.74–0.85)	40.6 (32.8–42.8) ^a,b^	93.4 (86.9–97.3)	6.2 (2.9–12.9)	0.6 (0.6–0.7)	90.0 (80.4–95.9)	51.8 (44.5–59.1)
IS1081-dPCR	5.5	0.72 (0.66–0.78)	27.1 (20.3–34.8)	93.4 (86.9–97.3)	4.1 (1.9–8.8)	0.8 (0.7–0.9)	85.7 (72.8–94.1)	46.7 (39.8–53.7)
Joint detection IS6110 & IS1081-dPCR	–	0.67 (0.60–0.73)	42.6 (34.7–50.8)	90.6 (83.3–95.4)	4.5 (2.4–8.4)	0.6 (0.5–0.7)	86.8 (77.1–93.5)	51.9 (44.4–59.3)
**Disseminated TB (16) compared with HC (*n*** **= 106)**								
IS6110-dPCR	7	0.98 (0.97–1.00)	100.0 (79.4–100.0) ^c,d^	93.4 (86.9–97.3)	15.1 (7.4–31.0)	0.0 (−)	69.6 (46.5–87.1)	100.0 (96.3–100.0)
IS1081-dPCR	5.5	0.95 (0.91–0.99)	68.8 (41.3–89.0) ^e,f^	93.4 (86.9–97.3)	10.4 (4.7–22.9)	0.3 (0.2–0.7)	61.1 (35.0–83.2)	95.2 (89.1–98.4)
**Extrapulmonary TB (56) compared with HC (*n*** **= 106)**								
IS6110-dPCR	7	0.83 (0.77–0.90)	50.0 (36.3–63.7)	93.4 (86.9–97.3)	7.6 (3.5–16.2)	0.5 (0.4–0.7)	80.0 (62.8–91.7)	78.0 (69.7–84.8)
IS1081-dPCR	5.5	0.81 (0.74–0.88)	39.3 (26.5–53.5)	93.4 (86.9–97.3)	4.2 (2.1–8.2)	0.7 (0.5–0.8)	68.7 (49.7–84.1)	73.8 (65.4–81.2)
**Bilateral TB (111) compared with HC (*n*** **= 106)**								
IS6110-dPCR	7	0.82 (0.76–0.87)	43.2 (33.9–53.0)	93.4 (86.9–97.3)	6.6 (3.1–13.8)	0.6 (0.5–0.7)	87.3 (75.4–94.8)	61.1 (53.1–68.7)
IS1081-dPCR	5.5	0.75 (0.68–0.81)	33.3 (24.7–42.9)	93.4 (86.9–97.3)	5.1 (2.4–10.8)	0.7 (0.6–0.8)	84.1 (69.7–93.4)	57.2 (49.5–64.7)

*AUC* area under receiver operating characteristic (ROC) curve, *LR+* positive likelihood ratio, *LR-* negative likelihood ratio, *PPV* positive predictive value, *NPV* negative predictive value

^a^ sensitivity comparison between IS6110- and IS1081- dPCR in total TB patients (*p* < 0.001, McNemar test)

^b^ sensitivity comparison between IS6110- and IS6110- or IS1081- positive dPCR in total TB patients (*p* = 0.250, McNemar test)

^c^ sensitivity comparison of IS6110-dPCR between Disseminated TB and Extrapulmonary TB patients (*p* < 0.001, Pearson Chi-Square test)

^d^ sensitivity comparison of IS6110-dPCR between Disseminated TB and Bilateral TB patients (*p* < 0.001, Pearson Chi-Square test)

^e^ sensitivity comparison of IS1081-dPCR between Disseminated TB and Extrapulmonary TB patients (*p* = 0.037, Pearson Chi-Square test)

^f^ sensitivity comparison of IS1081-dPCR between Disseminated TB and Bilateral TB patients (*p* = 0.006, Pearson Chi-Square test)

**Table 3:** Diagnostic performance of plasma IS6110- and IS1081- dPCR assay for Disseminated TB

	Cut-off point (copies/20 μl)	AUC	Sensitivity(%)	Specificity(%)	LR+	LR-	PPV(%)	NPV(%)
**Disseminated TB (16) compared with HC (*n*** **= 106)**								
IS6110-dPCR	16	0.98 (0.97–1.00)	77.8 (47.6–92.7)	99.1 (94.9–100.0)	86.1 (12.1–614.4)	0.2 (0.1–0.5)	92.9 (64.7–99.9)	97.2 (92.1–99.4)
IS1081-dPCR	14	0.95 (0.91–0.99)	37.5 (16.2–64.6)	99.1 (94.9–100.0)	39.8 (5.1–309.0)	0.6 (0.4–0.9)	85.7 (38.1–99.8)	91.3 (84.6–95.8)

*AUC* area under receiver operating characteristic (ROC) curve, *LR+* positive likelihood ratio, *LR-* negative likelihood ratio, *PPV* positive predictive value, *NPV* negative predictive value

### Sensitivity comparison of IS6110 & IS1081-dPCR with current TB diagnostic methods

Next, we compared the positive detection rates (sensitivity) of different TB diagnostic methods with joint detection of IS6110 and IS1081 by PCR (Table 4). All the TB patients in Table 4 were diagnosed by positive *M.tuberculosis* culture and/or smear test. Although the sensitivity of IS6110 & IS1081-dPCR was not promising as smear microscope (38.0% vs 93.5%) as well as mycobacterial culture (34.8% vs 97.0%), which was in accordance with a previous report that dPCR using total blood DNA could only detected 40% of TB cases [21]. On the other hand, the sensitivity of IS6110 & IS1081-dPCR was significantly higher than IS6110 qPCR (57.5% vs 22.5%) in 40 TB patients (*p* = 0.031). Furthermore, when we checked out the positive detection rate of IS6110 & IS1081-dPCR in smear-negative TB patients, there were 9 out of 19 plasma samples detected positive (47.4%), better than only IS6110- dPCR assay (42.1%), and especially higher than IS6110 qPCR (10%, 1/10, only 10 available qPCR results) (Table S2).

**Table 4:** Sensitivity comparisons of plasma IS6110 & IS1081-dPCR assay with routine diagnostic tests in diagnosis of TB

Methods	Number of TB Patients	Sensitivity	***p*** value
IS6110 & IS1081-dPCR vs Smear microscope	108	38.0% (41/108) vs 93.5% (101/108)	0.000
IS6110 & IS1081-dPCR vs Mycobacterial culture	66	34.8% (23/66) vs 97.0% (64/66)	0.000
IS6110 & IS1081-dPCR vs IS6110 qPCR	40	57.5% (23/40) vs 22.5% (9/40)	0.031

*qPCR* quantitative polymerase chain reaction; McNemar test was used to calculate *p* value

## Discussion

Digital PCR is a novel technology which can detect target sequences with relatively low abundance and obtain the absolute copy numbers of the targets. Our previous study has established IS6110 and IS1081 duplex dPCR system, by which the linear range of the two targets detecting *M.tuberculosis* derived DNA was 0.5–8733 and 0.2–2893 copies / μl reaction mixture and had a good reproducibility (r0.95) [22]. In the present study, we applied this duplex dPCR system to assess the accuracy of dPCR in TB diagnosis using more than 250 plasma samples from clinical specimens. To our knowledge, this is the first time applying IS1081 as one of the amplification targets to detect *M.tuberculosis* in dPCR assay.

Although IS6110 accounts for much more copies in the genome of *M.tuberculosis* than IS1081 (e.g. 16 copies vs. 6 copies in H37Rv), it has reported that the testing of IS6110 were not promising in clinical TB samples from several areas such as Southeast Asia and Vietnam, because some *M.tuberculosis* strains only had one copy of IS6110 in their genome [23]. Some clinical isolated strains were found to have no IS6110 element, which accounts for approximately 5% among the total isolates [24]. While study has shown that multiple copies of IS1081 were detected in the genome of *Mycobacterium tuberculosis* complex (MTBC) [25]. Consistently, our results also proved that 42 TB patients had higher IS1081 copies than IS6110. The recent Xpert MTB/RIF Ultra assay has been launched to improve the detection of *M.tuberculosis’* DNA in paucibacillary sputum by adding detection of IS6110 and IS1081 to rpoB [26]. However, the joint detection IS1081 & IS6110 -PCR did not significantly improve the diagnostic performance in total PTB under the current settings, and was not as promising as traditional smear microscope or mycobacterial culture which was in accordance with a previous report that dPCR using total blood DNA could only detected 40% of TB cases [21]. However, IS1081 & IS6110 -PCR has a higher sensitivity than IS6110 qPCR (*p* < 0.05) and was able to detect 47.4% of smear-negative TB patients. We do believe that it will make a difference when more samples are recruited from worldwide areas including Southeast Asia or Vietnam.

Recent studies have shown that dPCR had high accuracy and sensitivity in TB diagnosis [16–18]. When we recruited larger scale of samples (total of 155 PTB), the sensitivity and specificity of IS6110-dPCR were 40.6 and 93.4% respectively, equally promising as Ryota’s reported 65 and 93% (in 37 PTB). As bilateral lung distribution, extrapulmonary lesions and disseminated bacteria into blood were identified as personal factors for high copy number of *M.tuberculosis* derived DNA in plasma [16], we classified the TB patients into three different clinical types: bilateral TB, extrapulmonary TB and disseminated TB. The results showed that sensitivity of both IS6110- and IS1081- dPCR was the highest in patients with disseminated TB (IS6110, 100%; IS1081, 68.8%), while the sensitivity was a bit higher in patients with extrapulmonary TB (IS6110, 50.0%; IS1081, 39.3%) than that in bilateral TB (IS6110, 43.3%; IS1081, 33.3%), which was reasonable since there should be much more *M.tuberculosis* derived DNA segments in patients with disseminated phenotype than the other two types.

We also got positive dPCR results in some healthy controls that were clear of TB infection by T-SPOT. TB, but it was understandable because the sensitivity of T-SPOT. TB was about 90% according to a meta-analysis of newly reported evidence from 20 studies [27]. Furthermore, we and other groups have illustrated that there were *M.tuberculosis* nucleic acids in exosomes derived from plasma of both patients and healthy specimens as well as *M.tuberculosis* non-infected macrophages in vitro [28–30]. Considering the DNA extraction method we used here, the detected sequences in plasma may include bacterium genome DNA, circulating DNA as well as exosomal DNA. To this end, dPCR might be a more sensitive method to identify TB infecting people or those who has been infected by *M.tuberculosis*.

Recently, we have finished similar work on evaluation of dPCR in diagnostic accuracy analysis of cerebrospinal fluid for tuberculous meningitis and found that CSF IS6110-dPCR was a rapid and sensitive assay for TBM, which was even more sensitive than etiological tests currently used [15]. The results of our current study using plasma DNA as input for dPCR were not as outstanding as the former one, because the contents of CSF were much simpler and purer than plasma contributing to blood brain barrier. A recent study showed higher copy number of *M.tuberculosis* IS6110 elements were detected from plasma exosomes derived from TB patients although the sample sizes were relatively small (23 TB patients) [21]. Thus, plasma exosomes from more specimens in multicenter settings might be applied to evaluate the accuracy of dPCR platform in TB diagnosis.

## Conclusion

Our study evaluates the diagnostic potential of dPCR assay using plasma cell free DNA from a larger sample size of TB specimens and found that IS6110-dPCR is a rapid, moderate accurate and less invasive method to detect *M.tuberculosis* DNA in plasma of TB patients, and joint detection IS6110 & IS1081 -dPCR could improve the diagnostics of smear-negative TB. These results promotes the advances of the current reports related to the diagnosis performance of dPCR platforms using plasma and gives some concerns about using it as a diagnostic method in TB diagnosis.

## Supplementary information

**Additional file 1: Figure S1.** Representative original data of M.tb *IS6110*- and *IS1081*- dPCR in plasma samples. (a,b) Droplet dPCR amplification results of M.tb *IS6110* in three representative plasma samples of TB (a) and HC (b) with positive and negative controls. (c,d) Droplet dPCR amplification results of M.tb *IS1081* in three representative plasma samples of TB (c) and HC (d) with positive and negative controls. **Figure S2.** Agreement of two repeated tests for IS6110- and IS1081- dPCR. The correlation (Spearman correlation test) of copy number for duplicated *IS6110*-dPCR (a) and *IS1081*-dPCR (b), respectively. **Table S1** Diagnostic performance of joint detection IS6110 & IS1081-dPCR assay for TB. **Table S2** Sensitivity of IS6110 & IS1081-dPCR assay in detection of smear-negative TB.

## Data Availability

All the data and material related to this study are available from the corresponding author upon requirement.

## Abbreviations

TB: Tuberculosis
*M.tuberculosis*: Mycobacterial tuberculosis
MTBC: *Mycobacterium tuberculosis* complex
NATs: Nucleic acid amplification-based tests
LAMP: Loop-mediated isothermal amplification
PCR: Polymerase chain reaction
dPCR: Digital PCR
qPCR: Quantitative PCR
WHO: World health organization
CSF: Cerebrospinal fluid
TBM: Tuberculous meningitis
IS6110: Insert sequences 6110
IS1081: Insert sequences 1081
PTB: Pulmonary tuberculosis
HC: Healthy control
ROC: Receiver operating characteristics
AUC: Overall area under ROC curve
CI: Confidence interval
