# Supplementary material for: Evaluation of digital PCR assay in detection of M.tuberculosis IS6110 and IS1081 in tuberculosis patients plasma
Source: BMC Infect Dis. 2020 Sep 7;20:657. doi: 10.1186/s12879-020-05375-y (PMC7487892; doi:10.1186/s12879-020-05375-y)
Supplement: Supplementary file 1 — Additional file 1: Figure S1. Representative original data of M.tb IS6110- and IS1081- dPCR in plasma samples. (a,b) Droplet dPCR amplification results of M.tb IS6110 in three representative plasma samples of TB (a) and HC (b) with positive and negative controls. (c,d) Droplet dPCR amplification results of M.tb IS1081 in three representative plasma samples of TB (c) and HC (d) with positive and negative controls. Figure S2. Agreement of two repeated tests for IS6110- and IS1081- dPCR. The correlation (Spearman correlation test) of copy number for duplicated IS6110-dPCR (a) and IS1081-dPCR (b), respectively. Table S1 Diagnostic performance of joint detection IS6110 & IS1081-dPCR assay for TB. Table S2 Sensitivity of IS6110 & IS1081-dPCR assay in detection of smear-negative TB. [file 12879_2020_5375_MOESM1_ESM.docx]

**Supplementary materials**

**Supplementary Figure Legends**

**Figure S1. Representative original data of M.tb *IS6110*- and *IS1081*- dPCR in plasma samples.** (a,b) Droplet dPCR amplification results of M.tb *IS6110* in three representative plasma samples of TB (a) and HC (b) with positive and negative controls. (c,d) Droplet dPCR amplification results of M.tb *IS1081* in three representative plasma samples of TB (c) and HC (d) with positive and negative controls.

**Figure S2. Agreement of two repeated tests for IS6110- and IS1081- dPCR.** The correlation (Spearman correlation test) of copy number for duplicated *IS6110*-dPCR (a) and *IS1081*-dPCR (b), respectively.

**Supplementary Figures**

Figure S1


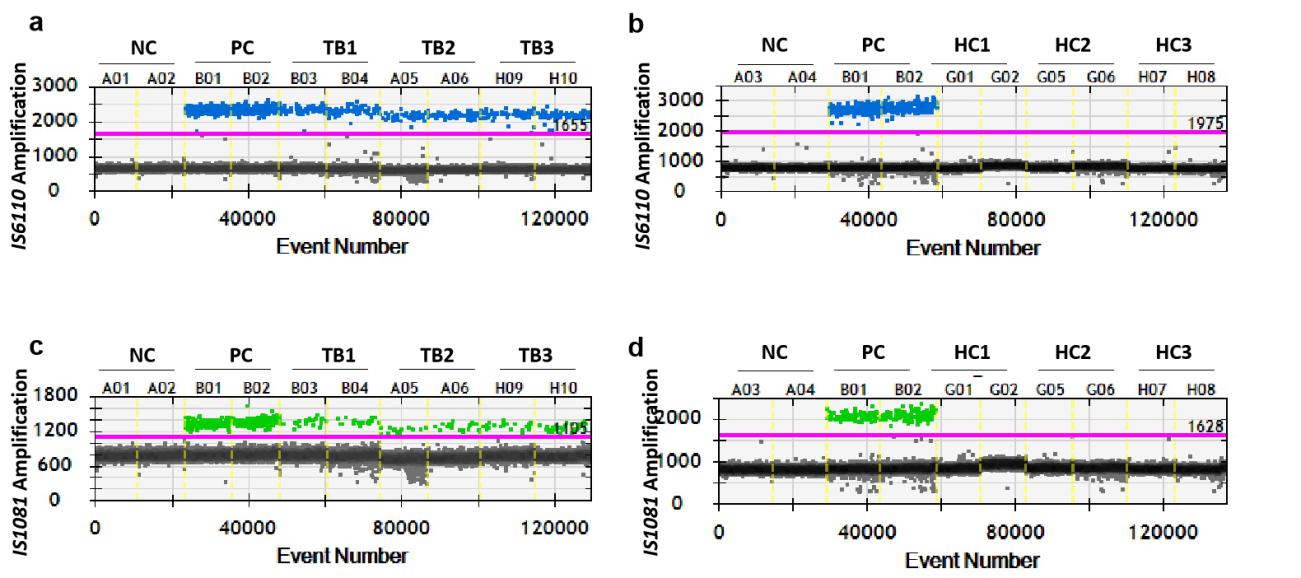


Figure S2


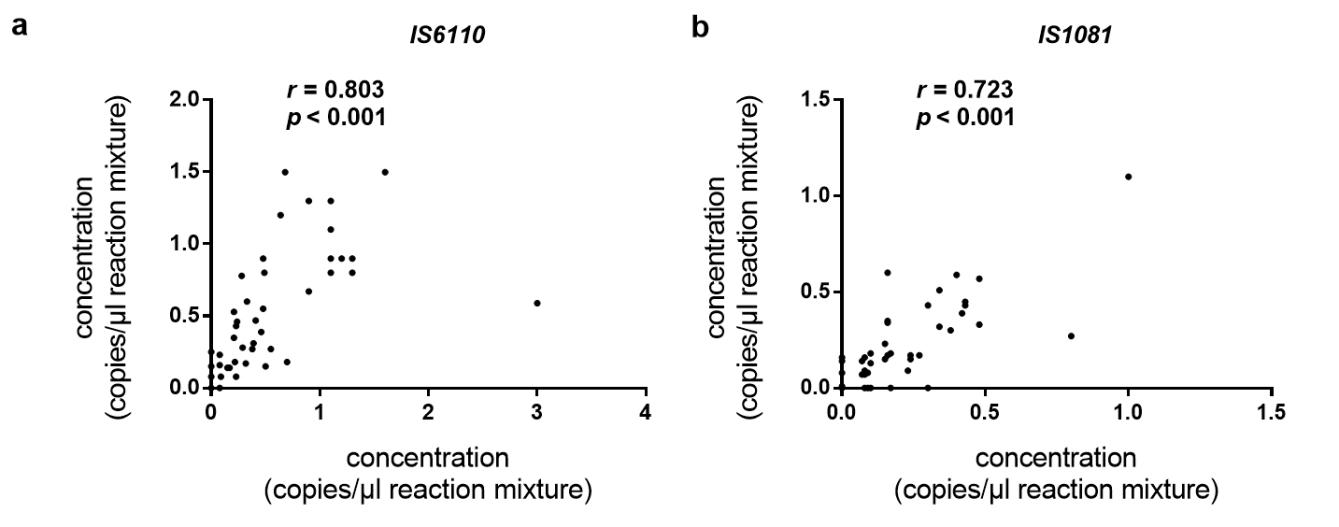


**Supplementary Table**

**Table S1 Diagnostic performance of joint detection IS6110 & IS1081-dPCR assay for TB**

|  | AUC | Sensitivity(%) | Specificity(%) |
| --- | --- | --- | --- |
| Disseminated TB (16) compared with HC (n=106) |  |  |  |
| Joint detection IS6110 & IS1081-dPCR | 0.95 (0.92-0.99) | 100.0 (80.5-100.0) | 90.6 (83.3-95.4) |
| Extrapulmonary TB (56) compared with HC (n=106) |  |  |  |
| Joint detection IS6110 & IS1081-dPCR | 0.71 (0.62-0.80) | 51.8  (38.0-65.3) | 90.6 (83.3-95.4) |
| Bilateral TB (111) compared with HC (n=106) |  |  |  |
| Joint detection IS6110 & IS1081-dPCR | 0.68 (0.61-0.75) | 45.9 (36.5-55.7) | 90.6 (83.3-95.4) |

**Table S2 Sensitivity of IS6110 & IS1081-dPCR assay in detection of smear-negative TB**

| **Methods** | **Number of Patients** | **Positive rate** |  |
| --- | --- | --- | --- |
| IS6110 & IS1081-dPCR | 19 | 47.4% (9/19) |  |
| IS6110-dPCR | 19 | 42.1%(8/19) |  |
| IS6110 qPCR | 10 | 10% (1/10) |  |
